# BEGEV as Salvage Therapy in Relapsed/Refractory Non-Hodgkin Lymphoma: Real-World Outcomes and Transplantation Feasibility

**DOI:** 10.3390/medicina62081502

**Published:** 2026-08-05

**Authors:** Ozlem Candan, Basak Buyukkurkcu, Sami Karti, Ant Uzay

**Affiliations:** 1 Department of Hematology, Kanuni Sultan Suleyman Training and Research Hospital, University of Health Sciences, 34303 Istanbul, Türkiye; 2 Department of Internal Medicine, Acıbadem University Atakent Hospital, 34303 Istanbul, Türkiye; buyukkurkcubasak@gmail.com; 3 Department of Hematology, Acıbadem University Atakent Hospital, 34303 Istanbul, Türkiye; sami.karti@acibadem.com (S.K.); ant.uzay@acibadem.com (A.U.)

**Keywords:** relapsed/refractory non-Hodgkin lymphoma, BEGEV, salvage therapy, stem cell transplantation, peripheral T-cell lymphoma

## Abstract

*Background and Objectives:* Relapsed/refractory non-Hodgkin lymphoma (R/R NHL) remains a major therapeutic challenge, particularly in patients with peripheral T-cell lymphomas (PTCL), who frequently exhibit poor responses to salvage therapy and inferior survival outcomes. Achieving adequate disease control before autologous stem cell transplantation (ASCT) is a critical determinant of long-term survival. The bendamustine, gemcitabine, vinorelbine, and prednisolone (BEGEV) regimen has demonstrated high efficacy and favorable tolerability in relapsed/refractory classical Hodgkin lymphoma; however, data regarding its role in NHL are extremely limited. *Materials and Methods:* We conducted a retrospective, single-center analysis of patients with R/R NHL who received the BEGEV regimen as salvage therapy. Clinical characteristics, response rates, transplantation outcomes, progression-free survival (PFS), overall survival (OS), and safety data were evaluated. Treatment responses were assessed according to standard radiologic response criteria. Survival analyses were performed using the Kaplan–Meier method. *Results:* A total of 68 patients with R/R NHL were included in the analysis. Diffuse large B-cell lymphoma (DLBCL) was the predominant histological subtype, accounting for 49 patients (72.1%). BEGEV was administered predominantly in later salvage settings. Response assessments were available for 56 patients. Among evaluable patients, the objective response rate (ORR) was 73.2%, whereas the ITT ORR was 60.3% (41/68). Following BEGEV therapy, 25 patients (36.8%) proceeded to autologous stem cell transplantation (ASCT), while 8 patients (11.8%) underwent allogeneic stem cell transplantation (allo-SCT). Exploratory analyses demonstrated longer overall survival (log-rank *p* = 0.012) and progression-free survival (log-rank *p* = 0.011) among patients who subsequently underwent transplantation; however, these findings should be interpreted with caution because of the retrospective study design and the potential for selection and immortal time biases. Median PFS and OS were 4 and 26 months, respectively. Adverse events were predominantly hematologic and generally manageable. *Conclusions:* BEGEV may represent a reasonable salvage regimen for selected patients with R/R NHL and may facilitate disease control allowing subsequent transplantation in selected patients, including selected PTCL patients.

## 1. Introduction

Relapsed or refractory (R/R) non-Hodgkin lymphoma (NHL) continues to represent a major therapeutic challenge despite substantial advances in frontline immunochemotherapy and transplantation strategies. Although salvage chemotherapy followed by autologous stem cell transplantation (ASCT) remains the standard treatment approach for transplant-eligible patients [1], outcomes remain suboptimal [2], particularly among those with primary refractory disease, early relapse, or biologically aggressive histologic subtypes. Achieving adequate disease control before ASCT has consistently been recognized as one of the most important prognostic factors associated with long-term survival in aggressive lymphomas [3]. However, evidence regarding the effectiveness of individual salvage regimens in the real-world setting for patients with relapsed/refractory NHL remains limited.

Several platinum- or ifosfamide-based salvage regimens, including ICE (ifosfamide, carboplatin, etoposide), DHAP (dexamethasone, cytarabine, cisplatin), GDP (gemcitabine, dexamethasone, cisplatin), and ESHAP (etoposide, methylprednisolone, cytarabine, cisplatin), have been extensively used in the management of R/R NHL [2,4]. Although these regimens can achieve meaningful response rates, they are frequently associated with considerable hematologic toxicity, infectious complications, hospitalization requirements, and inconsistent stem cell mobilization outcomes. In addition, no universally accepted salvage standard currently exists across the heterogeneous spectrum of NHL subtypes [5].

Peripheral T-cell lymphomas (PTCL) constitute a particularly aggressive subgroup of NHL characterized by poor prognosis, frequent chemoresistance, and inferior survival compared with aggressive B-cell lymphomas. Salvage treatment options for PTCL remain limited, and durable remissions are difficult to achieve even in transplant-eligible patients [6]. Consequently, there is an ongoing need for effective and tolerable salvage regimens capable of inducing rapid disease control while preserving transplant feasibility [7].

The BEGEV regimen, consisting of bendamustine, gemcitabine, vinorelbine, and prednisolone, was initially developed as a salvage strategy for relapsed/refractory classical Hodgkin lymphoma [8]. Importantly, several components of the regimen, particularly bendamustine- and gemcitabine-based combinations, have independently demonstrated meaningful activity in relapsed/refractory NHL across both B-cell and selected T-cell lymphoma subtypes [4,9,10]. In addition to antitumor efficacy, these approaches have attracted interest because of their relatively manageable toxicity profiles and potential feasibility before stem cell transplantation. Previous studies of BEGEV in Hodgkin lymphoma also demonstrated high complete response rates, favorable stem cell mobilization outcomes, and acceptable tolerability [8]. However, evidence regarding the efficacy of BEGEV specifically in NHL, particularly in PTCL, remains extremely limited.

Given the limited available data and the biological rationale supporting bendamustine- and gemcitabine-based salvage approaches, we retrospectively evaluated the real-world efficacy, safety, transplantation outcomes, and survival results of the BEGEV regimen in patients with relapsed/refractory NHL treated at our institution, including an exploratory analysis of PTCL patients.

## 2. Methods

### 2.1. Study Design and Patients

This retrospective single-center study included 68 consecutive adult patients with relapsed/refractory (R/R) NHL who received the BEGEV regimen between May 2018 and September 2025 as salvage therapy at our institution. Eligible patients had histologically confirmed NHL according to the World Health Organization classification and had experienced either relapsed or refractory disease following at least one prior line of systemic therapy. Clinical follow-up continued until death or the last available clinical assessment. Data were censored at the last follow-up in April 2026.

Clinical, pathological, and treatment-related data were retrospectively collected from institutional electronic medical records. Baseline characteristics included age, sex, Eastern Cooperative Oncology Group (ECOG) performance status, histologic subtype, Ann Arbor stage, bulky disease, extranodal involvement, bone marrow infiltration, lactate dehydrogenase (LDH) levels, International Prognostic Index (IPI), Prognostic Index for T-cell lymphoma (PIT), CD20 expression, ALK status, and double-hit/triple-hit biology when available.

### 2.2. Treatment Protocol

The BEGEV regimen consisted of bendamustine 90 mg/m^2^ on days 2 and 3, gemcitabine 800 mg/m^2^ on days 1 and 4, vinorelbine 20 mg/m^2^ on day 1, and prednisolone 100 mg on days 1–4. Treatment cycles were repeated every 21 days when clinically appropriate [8]. Among the 54 patients with CD20-positive B-cell lymphomas, rituximab administration was documented in 50 patients (92.6%). Rituximab administration could not be confirmed in the remaining four patients because of incomplete retrospective treatment records. Because the study also included T-cell lymphomas and other CD20-negative histologies, the regimen is referred to as BEGEV throughout the manuscript for consistency. Dose modifications were performed according to hematologic and non-hematologic toxicities. Supportive care included granulocyte colony-stimulating factor (G-CSF), antimicrobial prophylaxis, and transfusion support when clinically indicated. Hospitalization decisions were made at the discretion of the treating physician.

Patients achieving adequate disease control following salvage therapy were considered eligible for ASCT or allogeneic stem cell transplantation according to institutional transplantation criteria and overall clinical evaluation.

### 2.3. Response Assessment and Toxicity Evaluation

Treatment response was assessed using positron emission tomography/computed tomography (PET/CT) according to the Lugano 2014 classification criteria [11]. Responses were categorized as complete response (CR), partial response (PR), stable disease (SD), or progressive disease (PD).

Adverse events occurring during BEGEV administration were retrospectively evaluated and graded according to the Common Terminology Criteria for Adverse Events (CTCAE) version 5.0 (National Cancer Institute, 2017). Hematologic and non-hematologic toxicities were analyzed descriptively.

### 2.4. Study Endpoints

The primary endpoint of the study was the ORR, defined as the proportion of patients achieving CR or PR according to the Lugano 2014 classification criteria following BEGEV therapy. Secondary endpoints included CR and PR rates reported separately; PFS and OS, including median survival estimates and 12- and 24-month survival rates; transplantation feasibility and outcomes following autologous or allogeneic stem cell transplantation; and the incidence of grade ≥3 hematologic and infectious adverse events, including febrile neutropenia. Given the biological heterogeneity of NHL, exploratory subgroup analyses were performed for DLBCL, follicular lymphoma, and PTCL. Response outcomes were summarized separately for each subgroup. Separate survival estimates were provided for DLBCL, the predominant histological subtype, whereas survival analyses were not performed for the smaller follicular lymphoma and PTCL subgroups because of the limited sample sizes and number of outcome events. ORR and response component rates were summarized descriptively.

### 2.5. Statistical Analysis

Descriptive statistics were used to summarize baseline characteristics, treatment responses, transplantation outcomes, and toxicity data.

PFS was calculated from the initiation of BEGEV therapy to disease progression, relapse, or death from any cause. OS was calculated from BEGEV initiation until death from any cause or last follow-up. Survival curves were estimated using the Kaplan–Meier method and compared using the log-rank test. Median survival estimates were calculated using the Kaplan–Meier method. A *p*-value <0.05 was considered statistically significant. Statistical analyses were performed using IBM SPSS Statistics version 27.0 software (IBM Corp., Armonk, NY, USA).

### 2.6. Ethics Statement

This retrospective study was approved by the Acıbadem Mehmet Ali Aydınlar University Medical Research Ethics Committee (ATADEK) (Approval No: 2026-06/61; Date: 26 March 2026). The study was conducted in accordance with the principles of the Declaration of Helsinki. Due to the retrospective design of the study, the requirement for informed consent was waived by the ethics committee.

## 3. Results

A total of 68 patients with relapsed/refractory non-Hodgkin lymphoma (R/R NHL) treated with the BEGEV regimen were included in the analysis. The median age at BEGEV initiation was 55.5 years (range, 20–81), and 43 patients (63.2%) were male. Histologic subtypes were predominantly aggressive B-cell lymphomas, including diffuse large B-cell lymphoma (DLBCL) and high-grade B-cell lymphoma variants, followed by follicular lymphoma and peripheral T-cell lymphoma subtypes. Among patients with available data, most presented with advanced-stage disease, with stage III–IV disease documented in 41 of 57 evaluable patients (71.9%). Bulky disease was present in 27 of 59 evaluable patients (45.8%). ECOG performance status was 0–1 in 36 of 51 patients with available ECOG data (70.6%). Baseline demographic, clinical, and disease characteristics are summarized in Table 1.

Additional baseline disease characteristics included primary refractory disease, early relapse, CD20 expression, and double-hit/triple-hit biology. Primary refractory disease was documented in 38 patients (55.9%), while 24 patients (35.3%) experienced early relapse within 12 months of frontline therapy. CD20 expression was present in 54 patients (79.4%), whereas double-hit/triple-hit biology was identified in 35 patients (51.5%) among patients with available data.

Among patients with available prognostic data, most B-cell lymphoma patients had intermediate- or high-risk disease according to the International Prognostic Index (IPI). Treatment exposure before BEGEV initiation was heterogeneous and reflected the cohort’s heavily pretreated nature. Most patients had previously received anthracycline-based frontline immunochemotherapy, predominantly R-CHOP- or CHOEP-like regimens.

BEGEV was administered mainly in later salvage settings, most commonly as third-line therapy. Specifically, the regimen was used as second-line therapy in 1 patient (1.5%), third-line therapy in 53 patients (77.9%), fourth-line therapy in 11 patients (16.2%), and fifth-line or later therapy in 3 patients (4.4%). Previous salvage regimens most frequently included ESHAP-based approaches, followed by GEMOX-, DHAP-, and ICE-based regimens. A small number of patients had previously received bendamustine-containing therapies, lenalidomide-based regimens, brentuximab vedotin, radiotherapy, and prior autologous stem cell transplantation. Because 77.9% of patients received BEGEV as third-line therapy, comparative analyses according to treatment line were not considered statistically meaningful because of the marked imbalance between groups. Similarly, only four patients had received prior bendamustine-containing therapy before BEGEV; therefore, subgroup analysis according to prior bendamustine exposure was not considered statistically meaningful.

Response assessments were available for 56 of the 68 patients. Among evaluable patients, the ORR was 73.2% (41/56), including CR in 29 patients (51.8%) and PR in 12 patients (21.4%). SD and PD were observed in 5 (8.9%) and 10 (17.9%) evaluable patients, respectively. In an intention-to-treat (ITT) analysis including all enrolled patients, the ORR was 60.3% (41/68), with CR, PR, SD, and PD rates of 42.6%, 17.6%, 7.4%, and 14.7%, respectively. Response assessment was unavailable in the remaining 12 patients (17.6%) because of incomplete radiologic assessment or insufficient retrospective follow-up data. Detailed treatment response outcomes are summarized in Table 2.

Given the biological heterogeneity of NHL, an exploratory subgroup analysis was performed in patients with DLBCL, the predominant histological subtype in our cohort (n = 49). Response assessments were available for 41 DLBCL patients. Among evaluable patients, the ORR was 78.0% (32/41), including CR in 22 patients (53.7%) and PR in 10 patients (24.4%). In the ITT analysis including all DLBCL patients, the ORR was 65.3% (32/49). In the exploratory DLBCL subgroup, median PFS and OS were 4 and 25 months, respectively. The estimated 12- and 24-month OS rates were 83.5% and 51.0%, respectively.

An exploratory subgroup analysis was also performed in patients with follicular lymphoma (n = 10). Response assessments were available for eight patients. Among evaluable patients, the ORR was 62.5% (5/8), including CR in four patients (50.0%) and PR in one patient (12.5%). Three patients (37.5%) had progressive disease, while no patient had stable disease. In the ITT analysis including all patients with follicular lymphoma, the ORR was 50.0% (5/10).

An exploratory subgroup analysis was also performed in patients with PTCL (n = 5). Response assessments were available for three patients. Among evaluable patients, the ORR was 66.7% (2/3), including one complete response (33.3%) and one partial response (33.3%). One patient (33.3%) had stable disease. In the ITT analysis including all PTCL patients, the ORR was 40.0% (2/5).

**Figure 1:**
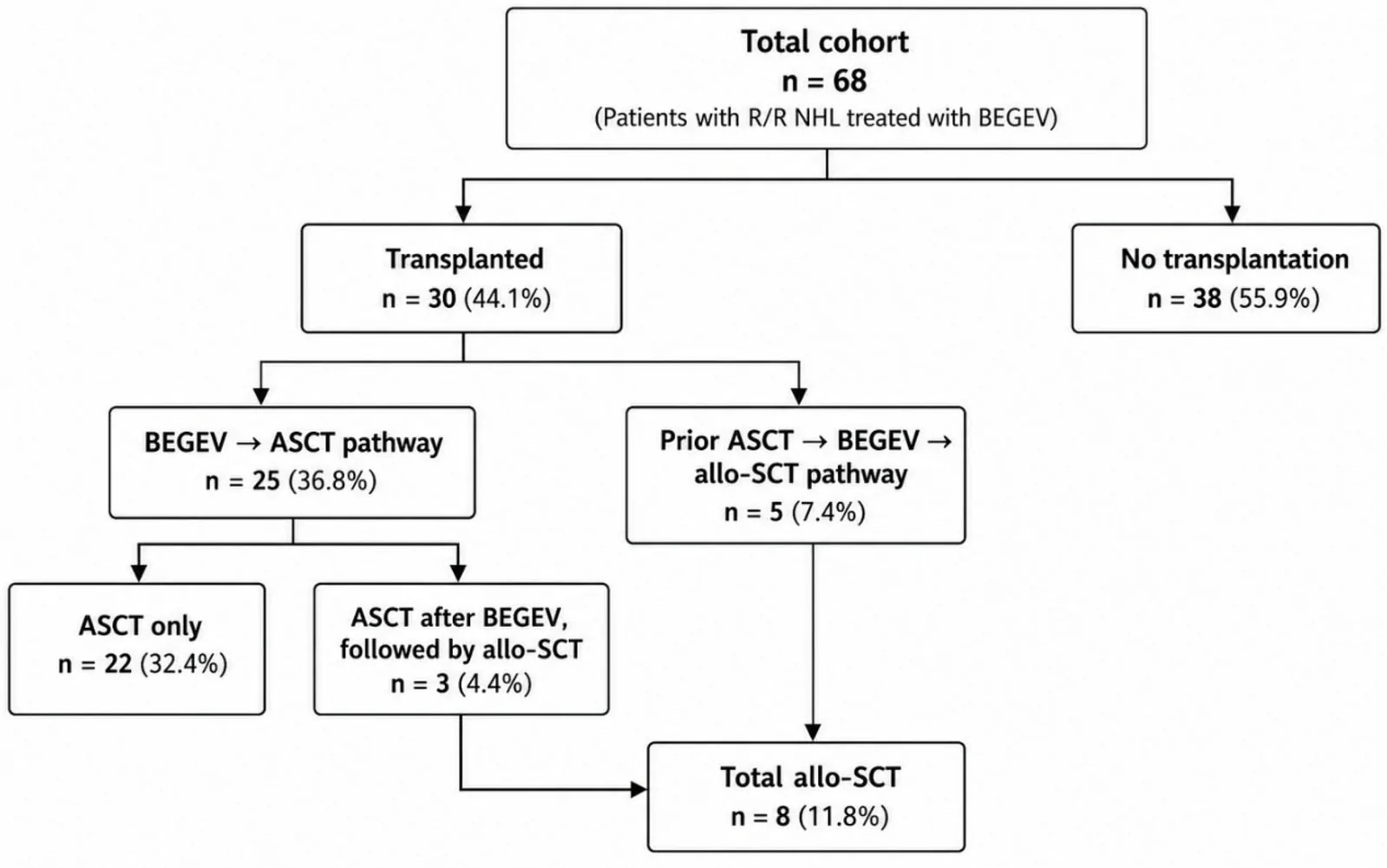
Patient flow and transplantation trajectories following BEGEV therapy in 68 patients with relapsed/refractory non-Hodgkin lymphoma (R/R NHL). Twenty-five patients subsequently underwent autologous stem cell transplantation (ASCT) following BEGEV, including 3 patients who subsequently proceeded to allogeneic stem cell transplantation (allo-SCT). An additional 5 patients with prior ASCT later underwent allo-SCT after BEGEV therapy. Overall, 8 patients underwent allo-SCT.

Following BEGEV therapy, 25 patients (36.8%) subsequently underwent autologous stem cell transplantation (ASCT). Subsequently, 8 patients (11.8%) underwent allogeneic stem cell transplantation, including several who had previously received ASCT. Overall, 30 unique patients proceeded to at least one transplantation procedure after BEGEV therapy. Detailed patient flow and transplantation trajectories following BEGEV therapy are illustrated in Figure 1.

**Figure 2:**
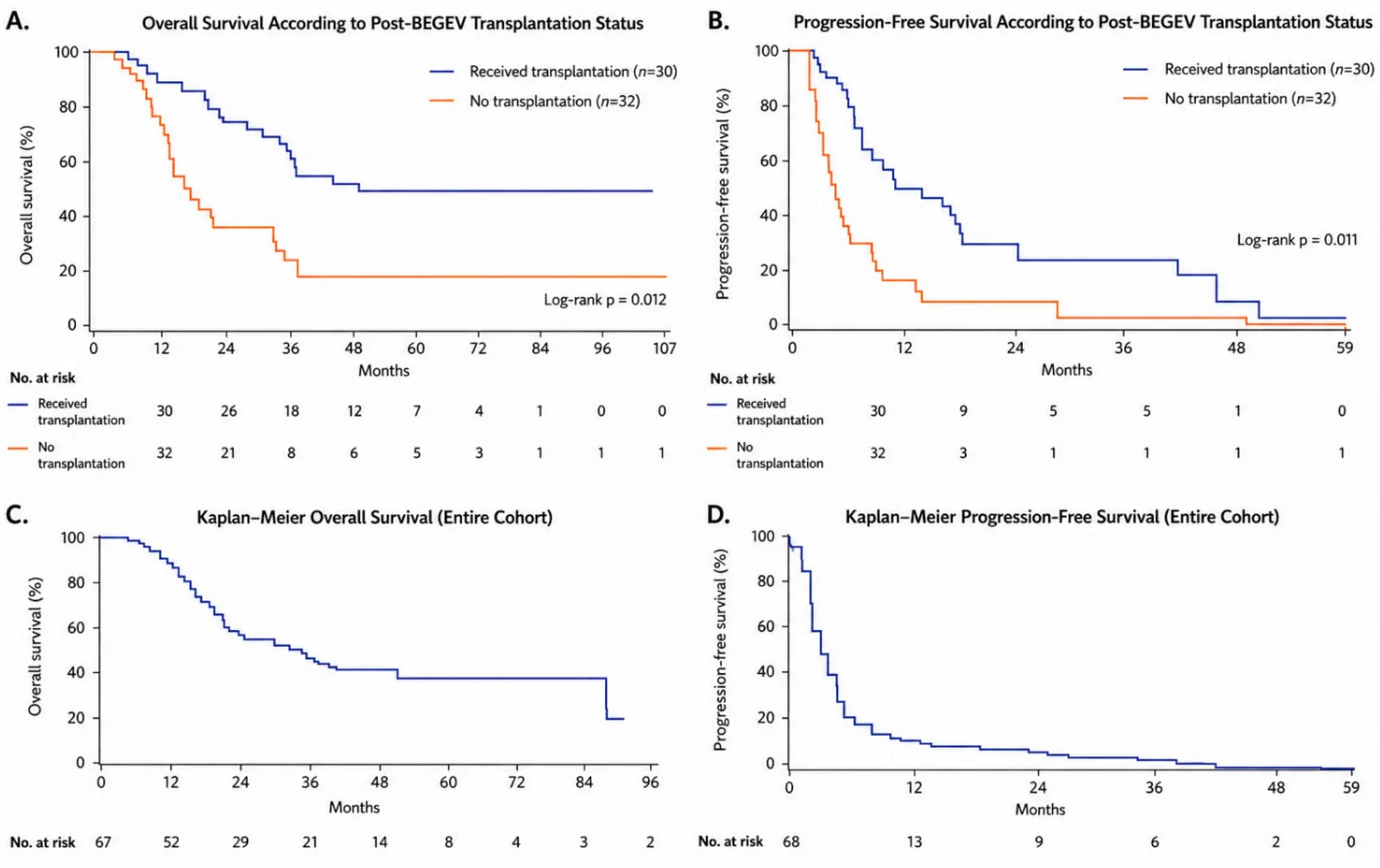
Kaplan–Meier survival analyses following BEGEV therapy. (**A**,**B**) Transplantation-stratified analyses were performed in the 62 patients with clearly documented transplantation status and follow-up data. (**C**) Overall survival analysis included 67 patients because survival status at the last follow-up was unavailable for one patient. (**D**) Progression-free survival analysis included all 68 patients.

Overall survival analyses included 67 patients because survival status at the last follow-up was unavailable for one patient owing to incomplete retrospective follow-up data, whereas progression-free survival analyses included all 68 patients. For survival analyses stratified by post-BEGEV transplantation status, patients with heterogeneous prior allo-SCT pathways or uncertain transplantation documentation were excluded to maintain analytical consistency. A total of six patients were excluded from these comparative analyses for these reasons. Consequently, transplantation-stratified survival analyses were performed in 62 evaluable patients with clearly documented transplantation status and follow-up data. Among these 62 patients, median PFS was 4 months and median OS was 26 months. The estimated 12- and 24-month OS rates were 81.5% and 52.8%, respectively, while the corresponding PFS rates were 17.6% and 13.2%, respectively. Exploratory survival analyses according to subsequent transplantation status demonstrated longer survival among transplanted patients; however, these findings should be interpreted cautiously because of the retrospective study design and the potential for selection and immortal time biases. Post-BEGEV transplantation was associated with superior overall survival (log-rank *p* = 0.012) and progression-free survival (log-rank *p* = 0.011) (Figure 2A,B). Kaplan–Meier analyses for overall survival (n = 67) and progression-free survival (n = 68) in the overall study cohort are presented in Figure 2C,D.

Treatment-related adverse events were predominantly hematologic and generally manageable. The most frequently observed toxicities included neutropenia, febrile neutropenia, anemia, thrombocytopenia, nausea/vomiting, mucositis, infectious complications, and fever. Febrile neutropenia and cytopenias represented the most clinically significant adverse events. Four deaths occurred during follow-up in temporal association with treatment. These events generally occurred in the setting of advanced progressive disease and infectious complications. However, due to the retrospective study design and the cohort’s advanced disease status, definitive attribution to treatment-related toxicity could not be established. Treatment-related adverse events are summarized in Table 3.

Overall, objective responses were observed in a substantial proportion of patients, and 30 patients subsequently proceeded to at least one transplantation procedure following BEGEV therapy. Treatment-related adverse events were predominantly hematologic and generally manageable.

## 4. Discussion

In this retrospective real-world study, the BEGEV regimen showed encouraging clinical activity with a manageable safety profile in patients with relapsed/refractory non-Hodgkin lymphoma treated across multiple lines of salvage therapy. Despite the heterogeneous and heavily pretreated nature of the cohort, BEGEV achieved a favorable overall response rate and allowed a substantial proportion of patients to proceed to stem cell transplantation. However, survival comparisons according to transplantation status should be interpreted cautiously because of potential selection and immortal time biases.

Salvage treatment in relapsed/refractory NHL remains challenging, particularly in patients with primary refractory disease, early relapse, aggressive histologic subtypes, or prior exposure to intensive chemotherapy. Although platinum- and ifosfamide-based regimens such as ICE, DHAP, GDP, and ESHAP continue to be widely used in clinical practice, these approaches are frequently associated with substantial hematologic toxicity, infectious complications, hospitalization requirements, and variable stem cell mobilization outcomes [12,13,14]. In this setting, bendamustine- and gemcitabine-containing combinations have attracted growing interest because of their relatively favorable balance between efficacy and tolerability. Nevertheless, clinical data specifically evaluating BEGEV in NHL remain extremely limited, particularly outside the Hodgkin lymphoma setting [14,15].

The efficacy outcomes observed in our cohort appear clinically relevant in light of the adverse baseline characteristics and heavily pretreated nature of the study population. Most patients had advanced-stage disease, extranodal involvement, elevated LDH levels, and multiple prior treatment exposures before BEGEV initiation. Despite these unfavorable features, BEGEV achieved an ORR exceeding 70%, with more than half of evaluable patients achieving complete remission. This response pattern compares favorably with several reported salvage approaches in R/R aggressive NHL, particularly considering that most patients in our cohort received BEGEV beyond the second-line setting [16,17]. Importantly, a substantial proportion of patients proceeded to transplantation, supporting the feasibility of BEGEV in patients proceeding to definitive consolidation therapy [18].

Although BEGEV was originally developed and has been most extensively studied in classical Hodgkin lymphoma, where it has consistently demonstrated high response rates and favorable tolerability before autologous stem cell transplantation [8], evidence supporting its use in relapsed/refractory NHL remains limited [12,13]. The present findings, together with the small number of previously published real-world studies, suggest that BEGEV may represent a reasonable salvage option in selected patients with relapsed or refractory NHL despite the greater biological heterogeneity of this disease. The clinical activity of BEGEV is likely attributable to the complementary mechanisms of its individual components. Bendamustine induces extensive DNA damage through its alkylating and purine analogue properties, gemcitabine inhibits DNA synthesis. The synergistic interaction of these agents provides substantial cytoreduction, which may explain the high response rates observed in both classical Hodgkin lymphoma and our heavily pretreated NHL cohort despite the relatively short progression-free survival [19,20]. Nevertheless, direct comparisons with other salvage regimens should be interpreted cautiously because of differences in patient characteristics, lymphoma subtypes, treatment lines, and study design.

The therapeutic landscape of relapsed/refractory NHL has evolved substantially with the introduction of novel treatment strategies, including CAR-T cell therapy, bispecific antibodies, and polatuzumab-based regimens, particularly for aggressive B-cell lymphomas [12,13,14,15,16,17]. These therapies have demonstrated promising efficacy and are increasingly incorporated into contemporary treatment algorithms. However, their use may be limited by patient eligibility, availability, reimbursement policies, cost, and the need for specialized treatment centers [12,13,14]. Consequently, conventional salvage chemotherapy continues to play an important role in routine clinical practice, particularly as a bridge to transplantation or for patients who are not candidates for these newer therapies [18]. Within this evolving treatment landscape, BEGEV may still represent a reasonable salvage option for carefully selected patients, particularly when bridging to transplantation is planned or access to novel therapies is limited.

Patients who ultimately underwent transplantation demonstrated longer survival. However, this observation should be interpreted cautiously because patients must survive and maintain sufficient clinical fitness to reach transplantation, introducing potential selection and immortal time biases. Therefore, no causal relationship between transplantation and improved survival can be inferred from the present analysis. Furthermore, landmark or time-dependent analyses may provide a more appropriate assessment of the association between transplantation and survival and should be considered in future studies. Nevertheless, the proportion of patients who were able to proceed to transplantation following BEGEV suggests that the regimen may provide effective disease control in selected patients. These findings are particularly relevant given that pre-transplant metabolic remission, especially PET negativity before ASCT, has consistently been associated with improved long-term survival outcomes in aggressive lymphomas [3,21,22].

Another clinically important aspect of our study was the inclusion of patients with PTCL, a subgroup historically associated with poor outcomes, frequent chemoresistance, and limited salvage treatment options. Although the PTCL cohort in our study was relatively small and exploratory in nature, several patients achieved clinically meaningful responses following BEGEV therapy. While definitive conclusions cannot be drawn because of the limited sample size and cohort heterogeneity, these observations may suggest potential activity of BEGEV in selected PTCL patients and support further investigation of bendamustine- and gemcitabine-based approaches in this particularly challenging disease setting [6,7,23].

The toxicity profile observed in our cohort was generally manageable and broadly consistent with the known safety characteristics of bendamustine- and gemcitabine-based salvage regimens [8,15,24,25]. Hematologic toxicities, particularly neutropenia and febrile neutropenia, represented the most clinically significant adverse events. Nevertheless, no unexpected toxicity signals were identified, and the overall safety profile appeared acceptable in the context of salvage therapy for aggressive lymphoma. Importantly, the regimen was successfully administered in routine clinical practice, and a substantial proportion of responding patients subsequently proceeded to transplantation.

## 5. Limitations

This study has several limitations, primarily related to its retrospective single-center design and the relatively heterogeneous patient population. The inclusion of multiple lymphoma subtypes and patients treated across different salvage lines may have influenced treatment outcomes and limited subgroup comparisons. In addition, the PTCL subgroup was relatively small, precluding definitive conclusions regarding efficacy in this setting.

Survival comparisons according to post-BEGEV transplantation status are particularly susceptible to immortal time bias, as patients must remain alive and progression-free long enough to undergo transplantation. Consequently, these analyses should be considered exploratory and hypothesis-generating rather than evidence of a causal survival benefit. Furthermore, landmark or time-dependent analyses were not performed because of the retrospective study design, limited sample size, and relatively small number of transplantation events, representing an additional methodological limitation of the present study.

Some important prognostic variables, including cell of origin (GCB vs. ABC), stem cell mobilization outcomes, and the exact number of BEGEV cycles, were not consistently available because of the retrospective nature of data collection and incomplete documentation. Furthermore, patients proceeding to transplantation likely represented a more favorable subgroup with better treatment response and performance status, which may have contributed to the observed survival advantage in transplanted patients. Finally, attribution of certain grade 5 events to treatment-related toxicity versus disease progression could not always be clearly distinguished during retrospective review. Similarly, the relatively small sample size, limited number of survival events, and heterogeneous study population precluded reliable multivariable Cox regression analyses; therefore, only univariate survival analyses were performed.

## 6. Conclusions

In conclusion, BEGEV may represent a reasonable salvage regimen for selected patients with relapsed/refractory NHL, demonstrating encouraging response rates and a manageable safety profile in this real-world cohort. A substantial proportion of patients were able to proceed to transplantation following BEGEV therapy, suggesting that the regimen may provide effective disease control in selected patients. In addition, the exploratory activity observed in PTCL patients is clinically noteworthy given the limited treatment options available in this setting.

Although prospective validation remains necessary, our findings provide real-world evidence supporting further evaluation of BEGEV as a salvage option in selected patients with B-cell and selected T-cell lymphomas and suggest that this regimen may represent a valuable salvage option in heavily pretreated patients.

## Figures and Tables

**Table 1:** Baseline Clinical and Disease Characteristics.

Variable	n (%) or Value
Median age, years (range)	55.5 (20–81)
Male sex	43 (63.2%)
ECOG 0–1 *	36/51 (70.6%)
Stage III–IV disease *	41/57 (71.9%)
Bulky disease *	27/59 (45.8%)
DLBCL	49 (72.1%)
Follicular lymphoma	10 (14.7%)
Peripheral T-cell lymphoma	5 (7.4%)
Mantle cell lymphoma	2 (2.9%)
Burkitt lymphoma	1 (1.5%)
Marginal zone/MALT lymphoma	1 (1.5%)
Second-line BEGEV	1 (1.5%)
Third-line BEGEV	53 (77.9%)
Fourth-line BEGEV	11 (16.2%)
Fifth-line or later BEGEV	3 (4.4%)

Abbreviations: ECOG, Eastern Cooperative Oncology Group; DLBCL, diffuse large B-cell lymphoma; MALT, mucosa-associated lymphoid tissue. Percentages marked with an asterisk (*) were calculated among evaluable patients with available data.

**Table 2:** Treatment Response Outcomes.

Outcome	Evaluable Patients (n = 56)	ITT Population (n = 68)
Overall response rate (ORR = CR + PR)	41 (73.2%)	41 (60.3%)
Complete response (CR)	29 (51.8%)	29 (42.6%)
Partial response (PR)	12 (21.4%)	12 (17.6%)
Stable disease (SD)	5 (8.9%)	5 (7.4%)
Progressive disease (PD)	10 (17.9%)	10 (14.7%)
Non-evaluable patients	—	12 (17.6%)

Abbreviations: ITT, intention-to-treat; ORR, overall response rate; CR, complete response; PR, partial response; SD, stable disease; PD, progressive disease.

**Table 3:** Adverse Events Observed During BEGEV Therapy (n = 68).

Adverse Event	Grade 1 n (%)	Grade 2 n (%)	Grade 3–4 n (%)	Grade 5 n (%)
Neutropenia	1 (1.5)	12 (17.6)	13 (19.1)	0
Febrile neutropenia	0	2 (2.9)	12 (17.6)	0
Anemia	1 (1.5)	6 (8.8)	5 (7.4)	0
Thrombocytopenia	1 (1.5)	6 (8.8)	3 (4.4)	0
Nausea/Vomiting	2 (2.9)	2 (2.9)	3 (4.4)	0
Mucositis	0	0	3 (4.4)	0
Infection	0	3 (4.4)	1 (1.5)	0
Fever	1 (1.5)	1 (1.5)	0	0
Fatal adverse events	0	0	0	4 (5.9)

Adverse events were graded according to the Common Terminology Criteria for Adverse Events, version 5.0 (CTCAE v5.0). Patients may have experienced multiple adverse events and/or more than one toxicity grade during treatment. Treatment-related attribution could not be definitively established for all grade 5 events because of the retrospective nature of the study.

## Data Availability

The datasets generated and/or analyzed during the current study are available from the corresponding author on reasonable request.
